# Single-cell RNA sequencing of a European and an African lymphoblastoid cell line

**DOI:** 10.1038/s41597-019-0116-4

**Published:** 2019-07-04

**Authors:** Daniel Osorio, Xue Yu, Peng Yu, Erchin Serpedin, James J. Cai

**Affiliations:** 1 Department of Veterinary Integrative Biosciences, Texas A&M University, College Station, TX 77843 USA; 2 Department of Veterinary Pathobiology, Texas A&M University, College Station, TX 77843 USA; 3 Department of Electrical and Computer Engineering, Texas A&M University, College Station, TX 77843 USA; 4 Interdisciplinary Program of Genetics, Texas A&M University, College Station, TX 77843 USA

**Keywords:** Gene expression, Gene expression analysis

## Abstract

In biomedical research, lymphoblastoid cell lines (LCLs), often established by *in vitro* infection of resting B cells with Epstein-Barr virus, are commonly used as surrogates for peripheral blood lymphocytes. Genomic and transcriptomic information on LCLs has been used to study the impact of genetic variation on gene expression in humans. Here we present single-cell RNA sequencing (scRNA-seq) data on GM12878 and GM18502—two LCLs derived from the blood of female donors of European and African ancestry, respectively. Cells from three samples (the two LCLs and a 1:1 mixture of the two) were prepared separately using a 10x Genomics Chromium Controller and deeply sequenced. The final dataset contained 7,045 cells from GM12878, 5,189 from GM18502, and 5,820 from the mixture, offering valuable information on single-cell gene expression in highly homogenous cell populations. This dataset is a suitable reference for population differentiation in gene expression at the single-cell level. Data from the mixture provide additional valuable information facilitating the development of statistical methods for data normalization and batch effect correction.

## Abstract (summary)

**Table:** 

Design Type(s)	transcription profiling design • strain comparison design
Measurement Type(s)	transcription profiling assay
Technology Type(s)	RNA sequencing
Factor Type(s)	ancestry status • sex
Sample Characteristic(s)	GM12878 cell • GM18502 cell • immortal human peripheral vein-derived B cell line cell

Machine-accessible metadata file describing the reported data (ISA-Tab format)

## Background & Summary

Immortalized cell lines are continuously growing cells derived from biological samples. Lymphoblastoid cell lines (LCLs) are one of the important members among many immortalized cell lines^1^. LCLs are usually established by infecting human peripheral blood lymphocytes *in vitro* with Epstein-Barr virus (EBV). The viral infection selectively immortalizes resting B cells, giving rise to an actively proliferating B cell population^2^. LCLs exhibit a low somatic mutation rate in continuous culture, making them the preferred choice of storage for individuals’ genetic material^3^. As one of the most reliable, inexpensive, and convenient sources of cells, LCLs have been used by several large-scale genomic DNA sequencing efforts such as the International HapMap and the 1,000 Genomes projects^4,5^, in which a large collection of LCLs were derived from individuals of different genetic backgrounds, to document the extensive genetic variation in human populations.

LCLs are also an *in vitro* model system for a variety of molecular and functional assays, contributing to studies in immunology, cellular biology, genetics, and other research areas^6–12^. It is also believed that gene expression in LCLs encompasses a wide range of metabolic pathways specific to individuals where the cells originated^13^. LCLs have been used in population-scale RNA sequencing projects^14–16^, as well as epigenomic projects^17^. For many LCLs used as reference strains, both genomic and transcriptomic information is available, making it possible to detect the correlation between genotype and expression level of genes and infer the potential causative function of genetic variants^18^. Furthermore, comparisons of gene expression profiles of LCLs between populations such as between Centre d’Etude du Polymorphisme Humain – Utah (CEPH/CEU) and Yoruba in Ibadan, Nigeria (YRI), have revealed the genetic basis underlying the differences in transcriptional activity between the two populations^16,19^.

With the advent of single-cell RNA sequencing (scRNA-seq) technology^20,21^, our approach for understanding the origin, global distribution, and functional consequences of gene expression variation is ready to be extended. For example, data generated from scRNA-seq provide an unprecedented resolution of the gene expression profiles at single cell level, which allows the identification of previously unknown subpopulations of cells and functional heterogeneity in a cell population^22–24^.

In this study, we used scRNA-seq to assess the gene expression across thousands of cells from two LCLs: GM12878 and GM18502. Cells were prepared using a Chromium Controller (10x Genomics, Pleasanton, CA) as described previously^21^ and sequenced using an Illumina Novaseq. 6000 sequencer. We present this dataset on the single-cell gene expression profile for more than 7,000 cells from GM12878 and more than 5,000 from GM18502. GM12878 is a popular sample that has been widely used in genomic studies. For example, it is one of three ‘Tier 1’ cell lines of the Encyclopedia of DNA Elements (ENCODE) project^17,25^. GM18502, derived from the donor of African ancestry, serves as a representative sample from the divergent population. The two cell lines are part of the International HapMap project, and genotypic information is available for both of them^4^. We also processed and sequenced an additional sample of 1:1 mixture of GM12878 and GM18502 using the same scRNA-seq procedure. Our dataset presented here provides a suitable reference for those researchers interested in performing between-populations comparisons in gene expression at the single-cell level, as well as for those developing new statistical methods and algorithms for scRNA-seq data analysis.

## Methods

### Cell culture

GM12878 and GM18502 cell lines were purchased from the Coriell Institute for Medical Research. Cells were cultured in the Roswell Park Memorial Institute (RPMI) Medium 1640 supplemented with 2mM L-glutamine and 20% of non-inactivated fetal bovine serum in T25 tissue culture flasks. Flasks with 20 mL medium were incubated on the upright position at 37 °C under 5% of carbon dioxide. Cell cultures were split every three days for maintenance. Note that authentication test and mycoplasm contamination screening on these freshly purchased cell lines were not undertaken in this study.

### Growth curve

Four culture flasks for each cell line were started with approximately 200,000 viable cells/mL to measure the growth rate of each cell line. Cells were prepared and cultured as described above. Viable cell number was estimated on a daily basis for four days. Briefly, 100 uL suspended cells from each flask were taken every day, to visualize the viable cells, the samples were stained using 10 uL of Trypan Blue (0.4%), and live cells were counted manually using a Neubauer counting chamber.

### Single cell preparation

Single-cell sample preparation was conducted according to Sample Preparation Demonstrated Protocol provided by 10x Genomics as follows: 1 mL of cell suspensions from each cell line (day 4, stable phase) was pelleted in Eppendorf tubes by centrifugation (400 g, 5 min). The supernatant was discarded, and the cells pellet was then resuspended in 1x PBS with 0.04% BSA, followed by two washing procedures by centrifugation (150 g, 3 min). After the second wash, cells were resuspended in ~500 uL 1x PBS with 0.04% BSA followed by gently pipetting mix 10–15 times. Cells were counted using an Invitrogen Countess automated cell counter (Thermo Fisher Scientific, Carlsbad, CA) and the viability of cells was assessed by Trypan Blue staining (0.4%).

### Generation of single cell GEMs (Gel bead in EMulsion) and sequencing libraries

Libraries were prepared using the 10x Genomics Chromium Controller in conjunction with the single-cell 3′ v2 kit. Briefly, the cell suspensions were diluted in nuclease-free water according to manufacturer instructions to achieve a targeted cell count of 5,000 for each cell line. The cDNA synthesis, barcoding, and library preparation were then carried out according to the manufacturer’s instructions. Libraries were sequenced in the North Texas Genome Centre facilities on a Novaseq. 6000 sequencer (Illumina, San Diego).

### Mapping of reads to transcripts and cells

Sample demultiplexing, barcode processing, and unique molecular identifiers (UMI) counting were performed by using the 10x Genomics pipeline CellRanger v.2.1.0 with default parameters. Specifically, for each library, raw reads were demultiplexed using the pipeline command ‘cellranger mkfastq’ in conjunction with ‘bcl2fastq’ (v2.17.1.14, Illumina) to produce two fastq files: the read 1 file contains 26-bp reads, each consists of a cell barcode and a unique molecule identifier (UMI), and the read 2 file contains 96-bp reads including cDNA sequences. Reads then were aligned to the human reference genome (GRCh38), filtered, and counted using ‘cellranger count’ to generate the gene-barcode matrix. Summary metrics of barcoding and sequencing from raw data are given in Table 1.

**Table 1:** Summary metrics for 10x Genomics scRNA-seq barcoding and sequencing of three LCL samples (GM12878, GM18502, and the 1:1 mixture).

	GM12878	GM18502	Mixture
Estimated Number of Cells	7,247	5,530	5,828
Mean Reads per Cell	65,466	91,493	83,326
Median Genes per Cell	2,954	3,960	3,621
Number of Reads	474,436,605	505,958,821	485,628,282
Valid Barcodes	97.20%	97.30%	97.20%
Sequencing Saturation	50.30%	53.50%	53.30%
Q30 Bases in Barcode	94.90%	94.80%	94.80%
Q30 Bases in RNA Read	90.20%	89.60%	89.90%
Q30 Bases in Sample Index	91.50%	93.40%	92.20%
Q30 Bases in UMI	94.80%	93.40%	94.70%
Reads Mapped to Genome	93.90%	93.70%	93.70%
Reads Mapped Confidently to Genome	92.00%	92.00%	92.00%
Reads Mapped Confidently to Intergenic Regions	2.60%	2.70%	2.70%
Reads Mapped Confidently to Intronic Regions	12.90%	13.10%	12.80%
Reads Mapped Confidently to Exonic Regions	76.50%	76.20%	76.50%
Reads Mapped Confidently to Transcriptome	72.60%	71.90%	72.50%
Reads Mapped Antisense to Gene	0.90%	0.90%	0.90%
Fraction Reads in Cells	90.70%	91.70%	89.80%
Total Genes Detected	21,329	20,701	21,151
Median UMI Counts per Cell	18,214	25,973	22,608

The estimates were produced by CellRanger on raw data, i.e., unfiltered feature-barcode matrix; values may differ slightly from those reported in the main text. For detailed definitions of metrics, refer to the 10x Genomics support website, https://support.10xgenomics.com/single-cell-gene-expression/software/pipelines/latest/output/gex-metrics.

### Quality control

Expression matrices were processed using Seurat (v2.3.4) R package^26^. Briefly, for each library, the expression matrix was loaded using the ‘Read10X’ function, and the default log-normalization was performed using the ‘NormalizeData’ function, followed by a cantering and scaling of the normalized values by using the ‘ScaleData’ function. Quality control (QC) measures, including UMI count, the number of genes detected per cell, and the percentage of mitochondrial transcripts were calculated. Cells with a proportion of mitochondrial reads lower than 10% and a library size smaller than 2.5x standard deviation (SD) from the average library size were considered good quality cells. The corresponding code used for the QC procedure is available online (see **Code availability**).

### Cell cycle phase and population assignment

Cell cycle phase assignment was made using the ‘CellCycleScoring’ function in the Seurat R package^26^, which uses the phase-specific marker genes, given by the ‘cc.genes’ dataset^27^. Cell population assignment, i.e., assigning cells in the mixture sample back to the cell line (GM12878 or GM18502) they belong to, was made using the Brunet algorithm^28^ for non-negative matrix factorization, in the NMF (v0.21) R package^29^. A set of marker genes (n = 252) with absolute log-fold change >2.5 identified by comparing the pure cell lines was used as inputs and the resulting probabilities after 2,000 iterations were used to assign each cell in the mixture to either GM12878 or GM18502.

### Dimensionality reduction

Expression matrices from GM12878, GM18502, and the mixture sample were merged and log-normalized using the function ‘MergeSeurat’. The resultant matrix was then centered and scaled. Highly variable genes were identified using function ‘FindVariableGenes’ in the Seurat R package^26^. Identified highly variable genes were used as input to produce the t-Distributed Stochastic Neighbour Embedding (t-SNE) projection using the ‘RunTSNE’ function with standard settings (perplexity = 30, theta = 0.5, maximum iteration = 1000, learning rate = 250, and momentum reduction = 0.5, by using the first 5 components from the principal component analysis). The Uniform Manifold Approximation and Projection (UMAP) was produced with the same set of highly variable genes as input using the function ‘RunUMAP’ with standard settings (min_dist = 0.3, metric = correlation, n_neighbors = 30).

### scRNA-seq versus bulk RNA-seq

For both GM12878 and GM18502, transcriptome has been previously sequenced using bulk RNA-seq. The availability of these existing data allowed us to examine the correlation between gene expression levels measured using scRNA-seq and bulk RNA-seq in the same LCLs. Thus, we downloaded the raw fastq files of bulk RNA-seq experiments from the Gene Expression Omnibus (GEO) database using accessions GSM484896^30,31^ (for GM12878) and GSM2392689^32,33^ (for GM18502) and quantified gene expression for both samples using Salmon^34^ (v0.12.0) against the human transcriptome (GRCh38). In addition, we also compared gene expression measured using scRNA-seq in GM12878 and GM18502 with the average gene expression measured in multiple samples from CEU and YRI populations. To do so, we downloaded the bulk RNA-seq data of 91 CEU and 89 YRI LCLs from the website of the Geuvadis RNA-seq project of 1,000 Genomes. The expression of each gene was measured as the mean of transcripts per million (TPM) values across all individuals of CEU or YRI population. To visualize the relationship of the single-cell gene-expression profiles of the two cell lines with their respective population, a principal component analysis (PCA) was performed. The input data for PCA was batch-effect corrected using the ‘removeBatchEffect’ function in the limma (3.4.0) R package^35^ and quantile normalized using the ‘normalize.quantiles’ function in the preprocessCore (1.46.0) R package.

## Data Records

The sequencing data from this study have been submitted as the BioProject reference (PRJNA508890), with descriptions of the Biosamples (SUB4895416, SUB4895422, SUB4895423). Raw data of three samples have been deposited at the National Center for Biotechnology Information (NCBI) Sequence Reads Archive (SRA) with accession ID: SRP172838^36^. For each sample, data include unprocessed scRNA-seq reads in two raw fastq files (*R1.fastq.gz for cell barcodes and UMIs, and *R2.fastq.gz for RNA reads), as well as an expression matrix file in matrix market exchange format (*.mtx) with columns corresponding to cells and row to genes. UMI matrices of this study have been deposited with the Gene Expression Omnibus at GEO: GSE126321^37^. The identifiers for the columns and rows are included in separated files (barcodes.tsv and genes.tsv). These processed files correspond to the output produced by the cell ranger pipeline. In addition, a supplementary table with the barcodes, population, UMI count, gene count, and mitochondrial transcript levels is included.

## Technical Validation

**Fig. 1:**
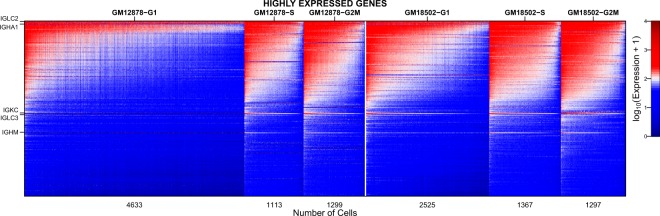
Heatmap of single-cell gene expression levels of the top 200 genes highly expressed in GM12878 and GM18502. Values are log-transformed UMI counts. For coloring purposes, values are truncated at a range between 0 and 4. Genes are arranged by the expression level. Cells are grouped according to cell cycle phases and sorted by their library size within each group. Immunoglobulin genes are labeled.

Here we present the scRNA-seq gene expression profile for 7,045 and 5,189 cells for GM12878 and GM18502, respectively. For GM12878, the median UMI counts per cell is 18,214 and the median number of genes detected (at least 1 UMI) per cell is 3,167; for GM18502, 25,973 and 3,891. Figure 1 is a heatmap of log-transformed expression data of top 200 highly expressed genes in the two LCLs. Cells are grouped by their cell cycle phases (G1, S, and G2/M) and sorted within each group by their library size. Among the top expressed genes, there are several immunoglobulin genes such as *IGLC2*, *IGHA1*, *IGKC*, *IGLC3*, and *IGHM*. These genes are not only expressed highly on average but also expressed highly variably across cells—i.e., highly expressed in one set of cells but no expression in another set of cells. We consider that this highly variable expression pattern can be attributed to immunoglobulin gene rearrangement. During the formation of the naïve-B cells, gene rearrangement process occurs to reshuffle different subunits of the variable (V), diversity (D) and joining (J) segments of immunoglobulin genes, resulting in the generation of a wide range of organism-specific antigen receptors that allow the immune system to recognize foreign molecules and initiate differential immune responses^38,39^. LCLs are produced through the rapid proliferation of few EBV-driven B cells from the blood cell population^40^. Thus, our scRNA-seq data of GM12878 and GM18502 offer a ‘snapshot’ of highly diverse immunoglobulin rearrangement profiles in a much larger population of polyclonal B cells found in the two donors.

**Fig. 2:**
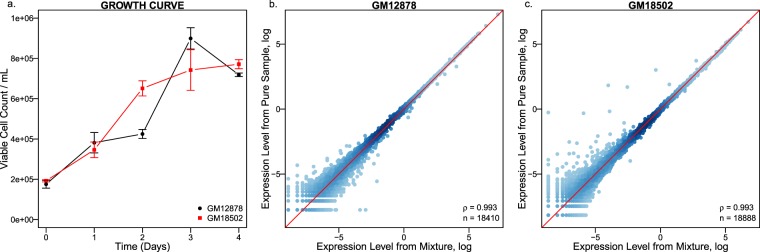
Cell growth curves and the gene expression correlations between samples. (**a**) Growth curve of the GM12878 and GM18502 cultured in the same RPMI 1640 medium. (**b**) Spearman’s correlation between the gene expressions profiles UMI average of the cells assigned to the CEU population from the mixture and those from the pure GM12878 cell line. Values were log-transformed, and each dot represents a gene. (**c**) Spearman’s correlation between the gene expression (average UMI) of cells assigned to the YRI population from the mixture and those from the pure GM18502 cell line. Values are log-transformed, and each dot represents a gene.

We also performed scRNA-seq with a 1:1 mixture sample of the two LCLs and obtained data for additional 5,820 cells with a median UMI counts per cell of 22,608 and a median number of genes detected per cell of 3,625. This mixture sample can be considered as a technical replicate for both GM12878 and GM18502. The use of the mixture sample facilitates direct comparison of gene expression between GM12878 and GM18502 because cells from two cell lines in the mixture were processed simultaneously in the same reaction, maximally eliminating the batch effect. We found that cells in the mixture were able to be assigned back to their original cell lines almost unambiguously using a non-negative matrix factorization algorithm (see Methods). Furthermore, the average gene expression measured in cells in the mixture, after discriminating cells in the mixture and assigning them to their respective one of original cell lines, was virtually indistinguishable from that measured in the original ‘pure’ cells (Fig. 2).

**Fig. 3:**
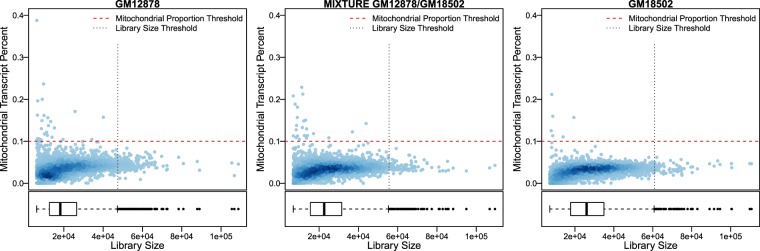
Distribution of the single-cell gene expression profiles under the defined quality control thresholds. There are 6,848 cells for the GM12878, 5,117 for the GM18502 and 5,710 for the mixture sample within the range of thresholds. These cells are considered to be of high quality.

The percentage of mitochondrial transcripts, an indicator of apoptotic cells, was computed for all cells sequenced in all the three samples. We found that no more than 0.4% of cells, that is, 26 cells from GM12878, 6 from GM18502, and 23 cells from the mixture sample, surpass the commonly used threshold of 10% mitochondrial transcripts^41^. This suggests that the majority of cells processed and sequenced were viable. Furthermore, as the 10x Genomics Chromium technology relies on droplets to partitioning cells and barcoding, it is normal some of them contain multiple cells in the cell droplet, making the estimation of the frequency of multiplets a critical aspect of quality control^42^. There are several ways to identify multiplets^43–45^. Here we adopted the threshold of 2.5x SD from the average library size for each cell. Based on this threshold, only 171 cells were considered to be multiplets for GM12878, 66 for GM18502, and 87 for the mixture (Fig. 3). These results support the quality of the dataset.

**Fig. 4:**
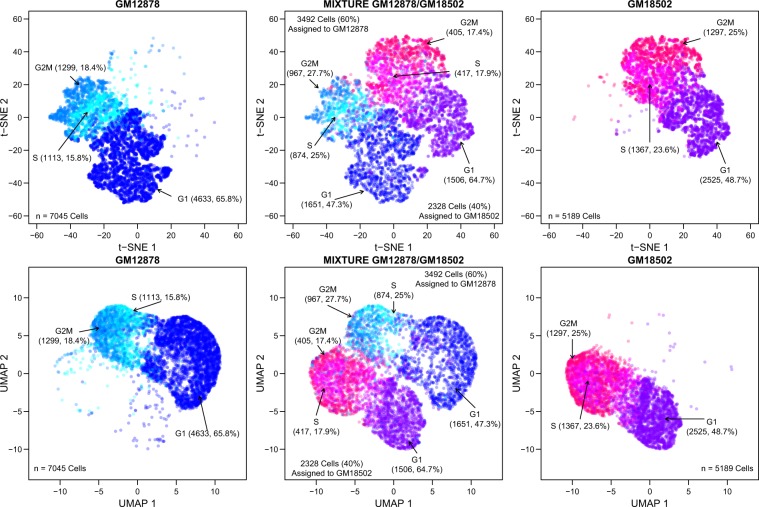
Plots of t-SNE and UMAP projections generated from the pooled scRNA-seq data of GM12878, GM18502, and the mixture samples. Separate panels are used to show cells labeled and colored differentially according to their cell line name and cell cycle state.

In either t-SNE or UMAP projection, no separation was observed between cells from the two pure cell lines, GM12878 and GM18502, and cells from the corresponding replicates of the two pure cell lines in the mixture (Fig. 4). This result suggests that cells in the mixture have the global expression profiles indistinguishable from those of cells of their original samples. Population signal of each sample allows a sample to be separated from others in the first two t-SNE or UMAP dimensional spaces. Furthermore, for each cell line, cells of different cell cycle phases are not entirely separated—a continuous path between the different clusters of cells exist. This allows researchers interested in cell cycle development to perform pseudo-time analysis^46^. Also, cells in the same cell cycle phase tend to be spread out and form a spectrum of cells in intermediate stages, indicating that cell proliferation is a continuous process and researchers interested in this process can use this dataset to refine reference cell sub-populations by their characterized expression profiles.

**Fig. 5:**
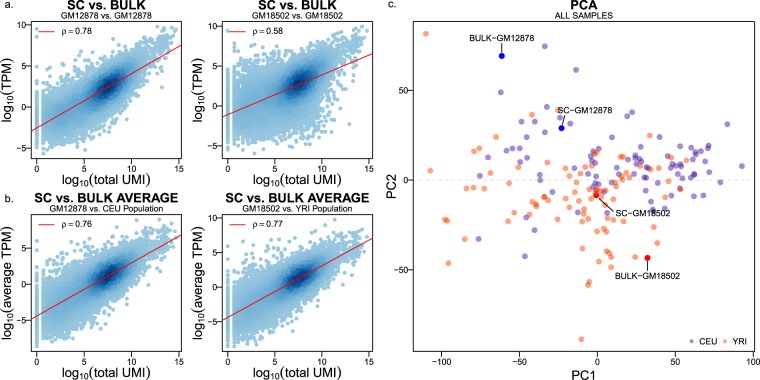
Gene expression correlations between single-cell sample, bulk-cell sample, and population average of bulk-cell samples. (**a**) Spearman’s correlation between the gene expressions profiles at the single-cell level and the bulk expression level (TPM) for GM12878 and GM18502. (**b**) Spearman’s correlation between the gene expressions profiles at the single-cell level for the GM12878 and GM18502 compared to the average bulk level expression (average TPM) for the available samples of CEU and YRI. Values are log-transformed, and each dot represents a gene. (**c**) PCA plot shows the similarity between the same samples’ gene expression profiles obtained using bulk RNA-seq and scRNA-seq.

For both GM12878 and GM18502, we conducted correlation analyses to validate our scRNA-seq expression data using bulk RNA-seq expression information as a reference. We first compared gene expression measured using scRNA-seq and bulk RNA-seq in the same LCL, GM12878 or GM18502. We also compared gene expression measured using scRNA-seq in GM12878 (and GM18502) with the average gene expression in corresponding population CEU (and YRI). We found that in all cases the correlations are highly significant and strong with Spearman correlation coefficients (SCCs) of 0.78, 0.58, 0.76, and 0.77, respectively (Fig. 5). Thus, when scRNA-seq data are pooled across cells, genes’ expression levels are largely recapitulated as they were measured using bulk RNA-seq. These results further support the quality of our scRNA-seq dataset. We note that the SCC (0.58) between GM18502 scRNA-seq and GM18502 bulk RNA-seq is lower than that (0.78) between GM12878 scRNA-seq and GM12878 bulk RNA-seq. This may be due to differences in cell population state at the time when GM18502 cells were harvested for scRNA-seq and bulk RNA-seq.

As long-lasting supplies of cells containing genotypic and phenotypic information matching that of B-cell origins, LCLs have contributed significantly to biomedical research. We present a high-quality dataset of scRNA-seq from homogenous cell populations of two LCLs, including GM12878—one of the most popular reference cell lines. Our dataset provides information that can be used to quantify cell-to-cell variability in gene expression and study cellular states and associated gene expression changes. It also informs the analysis and comparison of gene expression at the single-cell level between European and African LCLs. The data from the mixture sample are a suitable resource for estimating the technical variability of scRNA-seq and can also be used to calibrate statistical methods for data normalization and batch effect correction.

## ISA-Tab metadata file

Download metadata file

## Data Availability

All the required code to replicate the feature characterization of GM12878 or GM18502 and the mixture, as well as all figures included in this document, are available in a public repository on GitHub at https://github.com/cailab-tamu/sciData-LCL.

## References

[1] Nagy N (2017). Establishment of EBV-Infected Lymphoblastoid Cell Lines. Methods in Molecular Biology.

[2] Neitzel H (1986). A routine method for the establishment of permanent growing lymphoblastoid cell lines. Human Genetics.

[3] Mohyuddin A (2004). Genetic instability in EBV-transformed lymphoblastoid cell lines. Biochimica et Biophysica Acta (BBA).

[4] Durbin RM (2010). A map of human genome variation from population-scale sequencing. Nature.

[5] Sabeti PC (2007). Genome-wide detection and characterization of positive selection in human populations. Nature.

[6] Sie L, Loong S, Tan EK (2009). Utility of lymphoblastoid cell lines. Journal of Neuroscience Research.

[7] Hussain T, Mulherkar R (2012). Lymphoblastoid Cell lines: a Continuous *in Vitro* Source of Cells to Study Carcinogen Sensitivity and DNA Repair. *International*. Journal of Molecular and Cellular Medicine (IJMCM).

[8] Jiang S (2018). CRISPR/Cas9-Mediated Genome Editing in Epstein-Barr Virus-Transformed Lymphoblastoid B-Cell Lines. Current Protocols in Molecular Biology.

[9] Shim S-M (2012). MicroRNAs in human lymphoblastoid cell lines. Critical Reviews in Eukaryotic Gene Expression.

[10] Wheeler HE, Dolan ME (2012). Lymphoblastoid cell lines in pharmacogenomic discovery and clinical translation. Pharmacogenomics.

[11] Gurwitz D (2016). Human iPSC-derived neurons and lymphoblastoid cells for personalized medicine research in neuropsychiatric disorders. Dialogues in Clinical Neuroscience.

[12] Ansel A, Rosenzweig JP, Zisman PD, Melamed M, Gesundheit B (2016). Variation in Gene Expression in Autism Spectrum Disorders: An Extensive Review of Transcriptomic. Studies. Frontiers in Neuroscience.

[13] Amoli M, Carthy D, Platt H, Ollier W (2008). EBV Immortalization of human B lymphocytes separated from small volumes of cryo-preserved whole blood. International Journal of Epidemiology.

[14] Lappalainen T (2013). Transcriptome and genome sequencing uncovers functional variation in humans. Nature.

[15] Pickrell JK (2010). Understanding mechanisms underlying human gene expression variation with RNA sequencing. Nature.

[16] Martin AR (2014). Transcriptome Sequencing from Diverse Human Populations Reveals Differentiated Regulatory Architecture. PLoS Genetics.

[17] The, E. P. C. (2012). An integrated encyclopedia of DNA elements in the human genome. Nature.

[18] Sajantila A (2013). Editors’ pick: transcriptomes of 1000 genomes. Investigative Genetics.

[19] Stranger BE (2007). Population genomics of human gene expression. Nature Genetics.

[20] Tang F (2009). mRNA-Seq whole-transcriptome analysis of a single cell. Nature Methods.

[21] Zheng GXY (2017). Massively parallel digital transcriptional profiling of single cells. Nature Communications.

[22] Kolodziejczyk AA, Kim JK, Svensson V, Marioni JC, Teichmann SA (2015). The Technology and Biology of Single-Cell RNA Sequencing. Molecular Cell.

[23] Shalek AK (2013). Single-cell transcriptomics reveals bimodality in expression and splicing in immune cells. Nature.

[24] Marinov GK (2014). From single-cell to cell-pool transcriptomes: Stochasticity in gene expression and RNA splicing. Genome Research.

[25] Zhao B (2014). The NF-κB Genomic Landscape in Lymphoblastoid B Cells. Cell Reports.

[26] Satija R, Farrell JA, Gennert D, Schier AF, Regev A (2015). Spatial reconstruction of single-cell gene expression data. Nature Biotechnology.

[27] Tirosh I (2016). Dissecting the multicellular ecosystem of metastatic melanoma by single-cell RNA-seq. Science.

[28] Brunet JP, Tamayo P, Golub TR, Mesirov JP (2004). Metagenes and molecular pattern discovery using matrix factorization. Proceedings of the National Academy of Sciences.

[29] Gaujoux R, Seoighe C (2010). A flexible R package for nonnegative matrix factorization. BMC Bioinformatics.

[30] Kasowski M (2010). Variation in Transcription Factor Binding Among Humans. Science.

[31] Kasowski M (2009). Gene Expression Omnibus.

[32] Banovich NE (2018). Impact of regulatory variation across human iPSCs and differentiated cells. Genome Research.

[33] Banovich NE (2016). Gene Expression Omnibus.

[34] Patro R, Duggal G, Love MI, Irizarry RA, Kingsford C (2017). Salmon provides fast and bias-aware quantification of transcript expression. Nature Methods.

[35] Ritchie ME (2015). limma powers differential expression analyses for RNA-sequencing and microarray studies. Nucleic Acids Res.

[36] Osorio D, Xue Y, Yu P, Serpedin E, Cai J (2019). NCBI Sequence Read Archive.

[37] Osorio D, Xue Y, Yu P, Serpedin E, Cai J (2019). Gene Expression Omnibus.

[38] Papavasiliou F (1997). V(D)J recombination in mature B cells: a mechanism for altering antibody responses. Science.

[39] Tonegawa S (1983). Somatic generation of antibody diversity. Nature.

[40] Ryan JL (2006). Clonal evolution of lymphoblastoid cell lines. Laboratory Investigation.

[41] MacParland SA (2018). Single cell RNA sequencing of human liver reveals distinct intrahepatic macrophage populations. Nature Communications.

[42] Bloom JD (2018). Estimating the frequency of multiplets in single-cell RNA sequencing from cell-mixing experiments. PeerJ.

[43] McGinnis, C. S., Murrow, L. M. & Gartner, Z. J. DoubletFinder: Doublet detection in single-cell RNA sequencing data using artificial nearest neighbors. Preprint at, https://www.biorxiv.org/content/10.1101/352484v3 (2018). 10.1016/j.cels.2019.03.003 PMC6853612 30954475

[44] Wolock, S. L., Lopez, R. & Klein, A. M. Scrublet: computational identification of cell doublets in single-cell transcriptomic data. Preprint at, https://www.biorxiv.org/content/10.1101/357368v1 (2018). 10.1016/j.cels.2018.11.005 PMC6625319 30954476

[45] DePasquale, E. A. *et al*. DoubletDecon: cell-state aware removal of single-cell RNA-seq doublets. Preprint at, https://www.biorxiv.org/content/10.1101/364810v2 (2018).

[46] Trapnell C (2014). The dynamics and regulators of cell fate decisions are revealed by pseudotemporal ordering of single cells. Nature Biotechnology.

